# The cancer-testis lncRNA LINC01977 promotes HCC progression by interacting with RBM39 to prevent Notch2 ubiquitination

**DOI:** 10.1038/s41420-023-01459-1

**Published:** 2023-05-18

**Authors:** Anliang Xia, Qi Yue, Mingming Zhu, Jianbo Xu, Siyuan Liu, Yue Wu, Zhangding Wang, Zhu Xu, Hongda An, Qiang Wang, Shouyu Wang, Beicheng Sun

**Affiliations:** 1 Department of Hepatobiliary Surgery, Nanjing Drum Tower Hospital, Affiliated Hospital of Medical School, Nanjing University, Nanjing, China; 2 Nanjing Drum Tower Hospital, Clinical College of Jiangsu University, Nanjing, China; 3 Department of Hepatobiliary Surgery, The Affiliated Huai’an No.1 People’s Hospital of Nanjing Medical University, Huai’an, China; 4 Department of Gastroenterology, The Affiliated Drum Tower Hospital of Nanjing University Medical School, Nanjing, China; 5 Department of Hepatobiliary Surgery, The First Affiliated Hospital of Anhui Medical University, Hefei, China; 6 Jiangsu Key Laboratory of Molecular Medicine, Medical School of Nanjing University, Nanjing, China

**Keywords:** Cell growth, Hepatocellular carcinoma

## Abstract

Cancer-testis genes are involved in the occurrence and development of cancer, but the role of cancer-testis-associated lncRNAs (CT-lncRNAs) in hepatocellular carcinoma (HCC) remains to be explored. Here, we discovered a novel CT-lncRNA, LINC01977, based on the Genotype-Tissue Expression (GTEx) and The Cancer Genome Atlas (TCGA) databases. LINC01977 was exclusively expressed in testes and highly expressed in HCC. High LINC01977 levels correlated with poorer overall survival (OS) in individuals with HCC. Functional assays showed that LINC01977 promoted HCC growth and metastasis in vitro and in vivo. Mechanistically, LINC01977 directly bound to RBM39 to promote the further entry of Notch2 into the nucleus, thereby preventing the ubiquitination and degradation of Notch2. Furthermore, the RNA binding protein IGF2BP2, one of the m^6^A modification readers, enhanced the stability of LINC01977, resulting in its high level in HCC. Therefore, the data suggest that LINC01977 interacts with RBM39 and promotes the progression of HCC by inhibiting Notch2 ubiquitination and degradation, indicating that LINC01977 may be a potential biomarker and therapeutic target for HCC patients.

## Introduction

Hepatocellular carcinoma (HCC), the main type of primary liver cancer, is a common malignant tumor of the digestive system [1]. According to 2020 global cancer statistics, HCC ranks sixth in incidence and fourth in mortality among all cancer types [2]. HCC has the characteristics of insidious onset and lack of effective early diagnosis, which poses a serious threat to human health [3]. Despite great advances in the clinical treatment of HCC, including surgery, interventional therapy, and immunotherapy, the overall survival (OS) of HCC patients remains poor, as most patients are diagnosed at an advanced stage [4, 5]. The high metastasis rate and postoperative recurrence rate also pose challenges to the prognosis of HCC [6–8]. Therefore, in-depth exploration of the underlying molecular mechanisms of HCC progression and metastasis is urgently needed.

Cancer-testis (CT) genes, as the name suggests, are usually expressed only in testis tissue, with little or very low expression in other tissues [9]. However, CT genes are revitalized during malignancy and present abnormally high expression levels, which in turn drive the progression of malignancy [10]. The specific expression patterns of CT genes lay the foundation for them to be potential cancer driver genes, effective diagnostic markers, and ideal therapeutic targets [11–13]. Additionally, CT genes represent similarities between gametogenesis and tumorigenesis [9, 14]. Hence, a new pathogenesis of HCC may be elucidated, derived from the similarity between spermatogenesis and carcinogenesis as an entry point and the CT gene as a new breakthrough.

An increasing number of investigations have revealed that lncRNAs exert an irreplaceable role in different cancers [15–18]. The dynamic alterations of lncRNA expression are associated with tumorigenesis, cancer progression and metastasis [15–17]. Most lncRNAs play important functions in cancer by binding with proteins [19]. Recently, several researches have revealed that some lncRNAs are consistent with the characteristics of CT genes; that is, they are highly expressed in the tumors and specifically expressed in the testes [20–22]. For example, THOR and lnc-CTHCC, considered CT-lncRNAs, are closely related to melanoma and HCC carcinogenesis [21, 22]. In our previous studies, by constructing mouse lnc-CTHCC knockout models, it was clarified that lnc-CTHCC was involved in the occurrence and development of HCC by interacting with hnRNP K and activating YAP1 [22]. Therefore, CT-lncRNAs are beneficial to enrich the pathogenesis of HCC and provide potential therapeutic targets for HCC patients.

Here, we discovered another conserved CT-lncRNA, LINC01977, the function of which has not been reported in liver cancer. We found that CT-lncRNA LINC01977 played a key role in the IGF2BP2-LINC01977-RBM39-Notch2 axis, leading to the development of HCC.

## Results

### LINC01977 is a cancer-testis gene and correlates with poor prognosis in HCC

**Fig. 1:**
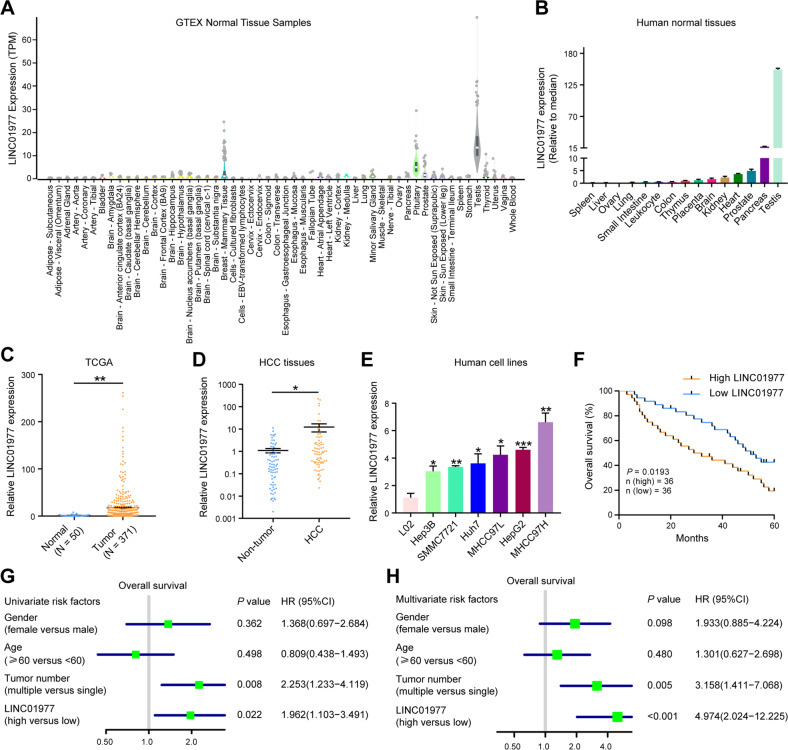
LINC01977 is a CT-lncRNA and is correlated with poor prognoses of patients with HCC. **A** Expression in TPM (transcripts per kilobase million) of LINC01977 among the GTEx normal tissue RNA-seq database. **B** The RNA levels of LINC01977 in human adult normal tissues. **C** The RNA levels of LINC01977 in HCC (*n* = 371) and normal tissues (*n* = 50) from the TCGA database. **D** The RNA levels of LINC01977 in HCC and paired nontumor tissues (*n* = 72). **E** The RNA levels of LINC01977 in HCC cell lines (Hep3B, SMMC7721, Huh7, MHCC97L, HepG2, and MHCC97H) and a normal liver cell line (L02). **F** Kaplan–Meier survival curves of OS in the HCC cohort (*n* = 72 individuals; *P* = 0.0193, log-rank test). **G** Univariate Cox regression analysis in the HCC cohort (*n* = 72 individuals). All bars correspond to 95% confidence intervals (95% CI). **H** Multivariate Cox regression analysis in the HCC cohort (*n* = 72 individuals). All bars correspond to the 95% CI. ^*^P < 0.05; ^**^P < 0.01; ^***^P < 0.001.

LINC01977 is a cancer-testis gene obtained from the GTEx and TCGA databases. In normal human tissues, LINC01977 is highly expressed in the testis, while its expression is very low or essentially absent in other normal tissues (Fig. 1A, B). Then, we evaluated the expression levels of 171 testis-specific non-coding RNAs in HCC from the TCGA database and discovered that LINC01977 showed a relative high expression level in HCC tissues compared to other testis-specific genes (Supplementary Fig. S1A, B). Also, we evaluated the expression of LINC01977 in tumor tissues and normal tissues through the TCGA database and discovered that LINC01977 showed a high expression level in HCC tissues (Fig. 1C and Supplementary Fig. S1C–E). The results were also confirmed to be higher in 72 HCC tissues (Fig. 1D). In addition to the tissue level, we also performed validation in common liver cancer cell lines. The data revealed that the expression of LINC01977 in liver cancer cell lines was significantly higher than that in immortalized normal hepatocyte L02 cells (Fig. 1E). Then, the coding potential of LINC01977 was investigated with the coding potential assessment tool, which indicated that LINC01977 is a non-coding RNA (Supplementary Fig. S1F). Also, the secondary structure of human LINC01977 was presented (Supplementary Fig. S1G). Therefore, these data show that LINC01977 is a cancer-testis lncRNA, namely, CT-lncRNA.

Furthermore, the relationship between LINC01977 expression and the prognosis of HCC patients was investigated. The results showed that high LINC01977 levels were correlated to reduced OS in HCC (Fig. 1F). We also explored the relationship between LINC01977 expression and the characteristics of 72 patients with HCC. The data revealed that individuals with high LINC01977 levels possessed greater tumor size (*P* = 0.026), more tumor number (*P* = 0.003), vascular invasion (*P* = 0.004), and advanced grade (*P* = 0.000) (Supplementary Table 1). Furthermore, LINC01977 was an independent risk factor for OS in HCC patients (hazard ratio (HR) = 4.974; 95% CI = 2.024–12.225) by univariate and multivariate Cox regression analyses (Fig. 1G, H). Therefore, these data reveal that LINC01977 may act as an independent prognostic factor for HCC patients.

### LINC01977 promotes HCC proliferation, angiogenesis and epithelial-mesenchymal transition (EMT) in vitro

**Fig. 2:**
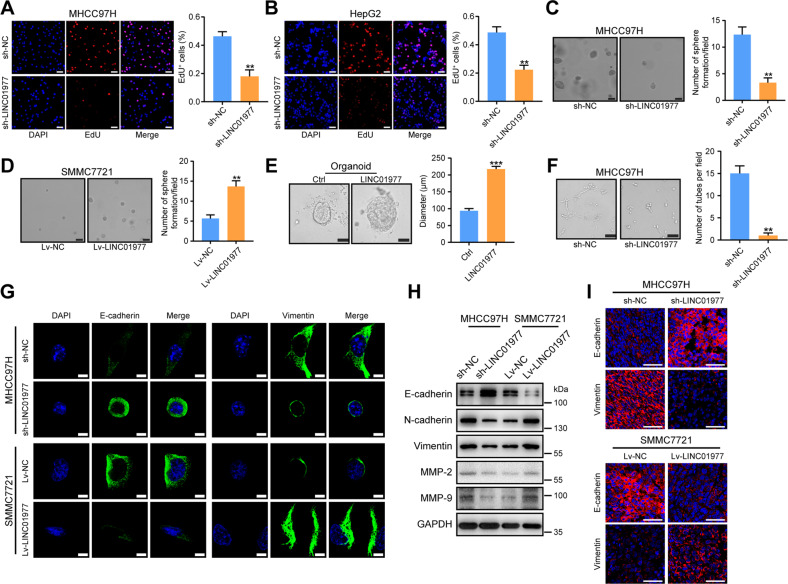
LINC01977 promotes HCC proliferation, angiogenesis and EMT in vitro. **A** EdU assays in MHCC97H cells with LINC01977 knockdown (scale bars = 50 μm). **B** EdU assays in HepG2 cells with LINC01977 knockdown (scale bars = 50 μm). **C** Soft agar assays in MHCC97H cells with LINC01977 knockdown (scale bars = 50 μm). **D** Soft agar assays in SMMC7721 cells overexpressing LINC01977 (scale bars = 50 μm). **E** Representative images of HCC organoids transfected with LINC01977 overexpression or control lentivirus for 2 weeks and quantification of organoid diameters (scale bars = 50 μm). **F** Angiogenesis assays in HUVECs cultured with medium from MHCC97H cells with LINC01977 knockdown (scale bars = 50 μm). **G** IF images of E-cadherin and Vimentin in HCC cells with the knockdown or overexpression of LINC01977 (scale bars = 10 μm). **H** Immunoblot images for E-cadherin, N-cadherin, Vimentin, Snail, MMP-2 and MMP-9 in HCC cells with the knockdown or overexpression of LINC01977. **I** IF images of E-cadherin and Vimentin in xenograft tumors with the knockdown or overexpression of LINC01977 (scale bars = 50 μm). ^**^P < 0.01; ^***^P < 0.001.

To investigate the biological function of LINC01977 in HCC, we first established stable LINC01977 knockdown cell lines (MHCC97H and HepG2) and LINC01977 overexpression cell lines (SMMC7721 and Hep3B) and verified the efficiencies of knockdown and overexpression using qRT-PCR (Supplementary Fig. S2A, B). The results of the clone formation assay showed that knockdown of LINC01977 significantly reduced the cell clone formation ability (Supplementary Fig. S2C). In contrast, the effect was enhanced upon overexpression of LINC01977 (Supplementary Fig. S2D). Additionally, EdU assays confirmed that LINC01977 had a significant effect on cell proliferation. Compared to controls, knockdown of LINC01977 reduced the number of EdU-positive cells in MHCC97H and HepG2 cells, while overexpression of LINC01977 increased the number of EdU-positive cells in SMMC7721 and Hep3B cells (Fig. 2A, B and Supplementary Fig. S2E, F). Furthermore, soft agar colony formation assays revealed that LINC01977 favors spheroid formation efficiency (Fig. 2C, D). HCC organoid models also confirmed that overexpression of LINC01977 by lentiviral infection promoted HCC organoid growth (Fig. 2E). Additionally, angiogenesis experiments showed that conditioned medium from LINC01977-deficient MHCC97H cells greatly decreased the tube formation of HUVECs compared with controls (Fig. 2F).

To further evaluate the function of LINC01977 in HCC metastasis, metastasis-related assays were carried out. First, Transwell experiments confirmed that cell migration and invasion were significantly decreased after knockdown of LINC01977 in MHCC97H and HepG2 cells (Supplementary Fig. S3A, B). In contrast, after overexpression of LINC01977 in SMMC7721 and Hep3B cells, the migrating and invading cells were obviously increased (Supplementary Fig. S3C, D). EMT plays a key role in HCC metastasis. IF staining showed that knockdown of LINC01977 reduced the expression level of Vimentin and enhanced the expression level of E-cadherin in MHCC97H cells (Fig. 2G). Overexpression of LINC01977 increased the expression level of Vimentin and reduced the expression level of E-cadherin in SMMC7721 cells (Fig. 2G). The western blotting results validated that LINC01977 promoted EMT (Fig. 2H). Additionally, further analysis of mouse subcutaneous tumor tissues also confirmed that LINC01977 contributed to the occurrence of EMT (Fig. 2I). The results also confirmed that LINC01977 contributed to the occurrence of EMT. Overall, these results suggest that LINC01977 promotes HCC proliferation, angiogenesis and EMT in vitro.

### LINC01977 promotes HCC growth and metastasis in vivo

**Fig. 3:**
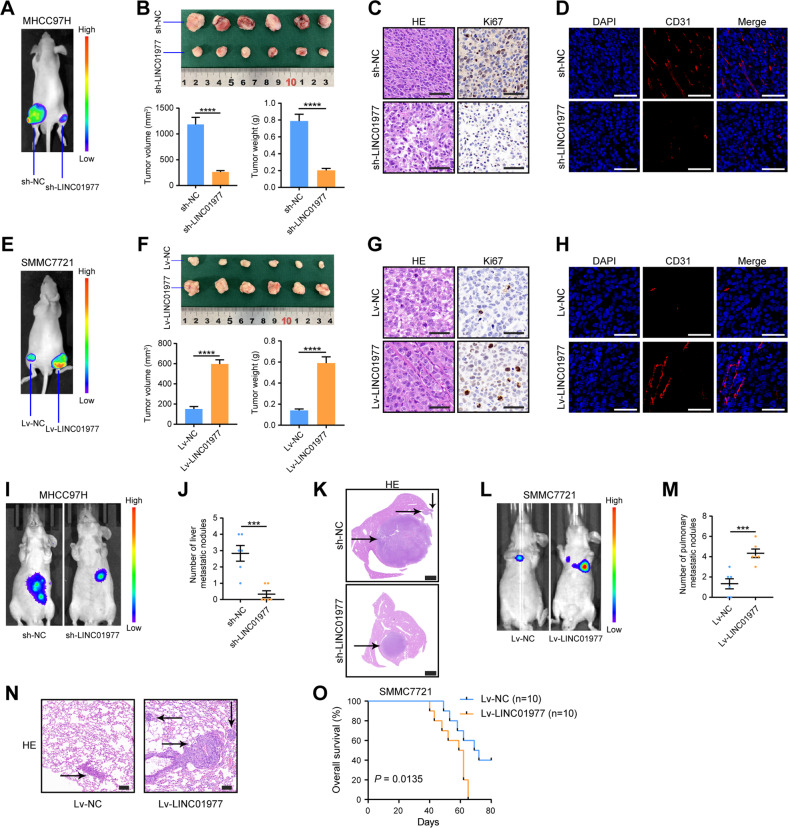
LINC01977 promotes HCC growth and metastasis in vivo. **A**–**D** Knockdown of LINC01977 significantly inhibited HCC proliferation in nude mice (*n* = 6). Representative bioluminescent images of subcutaneous tumors (**A**) and gross morphology from subcutaneous tumors, tumor volume and tumor weight (**B**) are shown. **C**, **D** HE, Ki67 and CD31 staining in xenograft tumors is also depicted (scale bars = 50 μm). **E**–**H** Overexpression of LINC01977 significantly promoted HCC proliferation in nude mice (*n* = 6). Representative bioluminescent images of subcutaneous tumors (**E**) and gross morphology from subcutaneous tumors, tumor volume and tumor weight (**F**) are shown. (**G**, **H**) HE, Ki67 and CD31 staining in xenograft tumors are also depicted (scale bars = 50 μm). **I**–**K** Knockdown of LINC01977 significantly inhibited HCC intrahepatic metastasis in nude mice (*n* = 6). Representative bioluminescent images in liver regions (**I**), quantification of the metastatic nodes (**J**) and HE-stained liver sections (**K**) are shown. The metastatic lesions are indicated by black arrows (**K**) (scale bars = 2000 μm). **L**–**N** Overexpression of LINC01977 significantly promoted HCC lung metastasis in nude mice (*n* = 6). Representative bioluminescent images in lung regions (**L**), quantification of the metastatic nodes (**M**) and HE-stained lung sections (**N**) are shown. The metastatic lesions are indicated by black arrows (**N**) (scale bars = 100 μm). **O** Kaplan–Meier survival curves of OS between mice injected with SMMC7721-Lv-NC and those injected with SMMC7721-Lv-LINC01977 (*n* = 10 mice per group; *P* = 0.0135, log-rank test). ^***^P < 0.001; ^****^P < 0.0001.

To further confirm the role of LINC01977 in vivo, the subcutaneous tumor models were established. Bioluminescence imaging revealed that luciferase activity was significantly reduced in tumors with LINC01977 knockdown compared with controls (Fig. 3A). Knockdown of LINC01977 delayed tumor growth, as reflected in tumor size, tumor weight, HE and Ki67 staining (Fig. 3B, C). Additionally, CD31 IF staining revealed that knockdown of LINC01977 reduced microvessel density, which was consistent with the in vitro angiogenesis assay (Fig. 3D). Furthermore, the role of LINC01977 overexpression on tumorigenicity was further investigated. The results indicated that overexpression of LINC01977 accelerated tumor growth, as revealed by bioluminescence imaging, tumor size and tumor weight (Fig. 3E, F). Both Ki67 and CD31 staining uncovered that overexpression of LINC01977 favored accelerated cell proliferation and increased microvessel density (Fig. 3G, H).

Then, in vivo metastasis assays were performed using nude mice. First, mouse models of surgical orthotopic implantation were established. Liver orthotopic implantation was established with LINC01977-knockdown subcutaneous tumors and control tumors. The MHCC97H knockdown group had weaker bioluminescence intensity and fewer liver metastases (Fig. 3I, J). HE staining also verified that the liver metastases in the LINC01977 knockdown group were significantly decreased (Fig. 3K), indicating that knockdown of LINC01977 suppressed intrahepatic metastasis. Moreover, lung metastasis models were also established. Tail vein injection of nude mice was performed with SMMC7721 cells overexpressing LINC01977 and control cells. The LINC01977-overexpressing group had greater bioluminescence intensity and a greater number of pulmonary metastatic nodules (Fig. 3L–N). Overexpression of LINC01977 promoted lung metastasis. In addition, the OS time of nude mice in the LINC01977 overexpression group was significantly lower than that in the control group (Fig. 3O). Therefore, all the data suggest that LINC01977 promotes HCC growth and metastasis in vivo.

### LINC01977 binds to RBM39 and promotes HCC tumorigenesis and progression

**Fig. 4:**
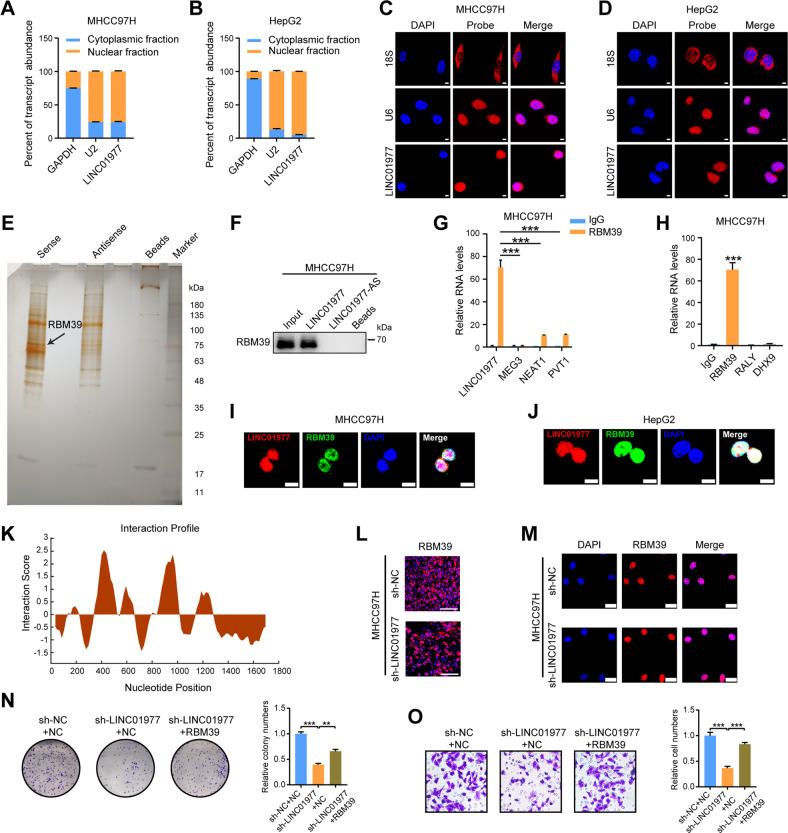
LINC01977 interacts with RBM39, and RBM39 mediates LINC01977-induced progression of HCC. **A**, **B** Nucleocytoplasmic separation assays of LINC01977 in MHCC97H and HepG2 cells. Cytosolic and nuclear markers include GAPDH and U2. **C**, **D** FISH of LINC01977 in MHCC97H and HepG2 cells (scale bars = 5 μm). Cytosolic and nuclear markers include 18 S and U6. **E** Representative image of silver staining for the LINC01977-protein complex. The black arrow indicates the additional band that was present when the cell lysates were incubated with LINC01977 compared with antisense RNA. **F** Western blotting assays of the specific interaction of LINC01977 with RBM39. **G**, **H** RIP assays showing the interaction of RBM39 with LINC01977 in MHCC97H cells. Abundant nuclear lncRNA control (MEG3, NEAT1 and PVT1) and RNA-binding protein controls (RALY and DHX9) are shown. **I**, **J** Confocal images showing colocalization of LINC01977 (red) and RBM39 (green) in MHCC97H and HepG2 cells (scale bars = 10 μm). **K** The potential binding regions between LINC01977 and RBM39 by the catRAPID database (http://service.tartaglialab.com/page/catrapid_group). **L** IF staining of RBM39 in xenograft tumors with the knockdown of LINC01977 (scale bars = 50 μm). **M** IF staining of RBM39 in MHCC97H cells with LINC01977 knockdown (scale bars = 25 μm). **N** Overexpression of RBM39 rescued the colony formation ability of MHCC97H cells with LINC01977 knockdown. **O** Overexpression of RBM39 rescued the invasion ability of MHCC97H cells with LINC01977 knockdown. ^**^P < 0.01; ^***^P < 0.001.

To better clarify the molecular mechanism of LINC01977 in HCC, the subcellular location of LINC01977 was detected. LINC01977 was confirmed to be predominantly located in the nucleus by nucleocytoplasmic separation assays (Fig. 4A, B). Consistently, fluorescence in situ hybridization (FISH) analyses also verified this phenomenon (Fig. 4C, D). Since lncRNAs usually bind to proteins to exert relevant biological functions, RNA pulldown and mass spectrometry analyses were performed using biotinylated LINC01977 and MHCC97H cell lysates. The results showed that LINC01977 bound to the RBM39 protein (Fig. 4E, F). Furthermore, RNA immunoprecipitation (RIP) assays were performed to further verify the specific binding of LINC01977 and RBM39. The data showed that RBM39 significantly bound to LINC01977 but not to other abundant nuclear lncRNAs such as *MEG3*, *NEAT1* and *PVT1* (Fig. 4G). Compared to RBM39, putative nuclear RNA-binding proteins, such as RALY and DHX9, did not bind to LINC01977 (Fig. 4H). FISH and IF assays together confirmed that LINC01977 and RBM39 colocalized in the nucleus from MHCC97H and HepG2 cells (Fig. 4I, J). Also, the possible binding sites between LINC01977 and RBM39 were predicted using the catRAPID database (http://service.tartaglialab.com/page/catrapid_group). We found that nucleotides 959–1030 of LINC01977 and amino acids 301–352 of RBM39 were the most likely binding regions (Fig. 4K).

Moreover, RBM39, a known RNA-binding protein, showed a higher expression level in HCC from TCGA data (Supplementary Fig. S4A). Using our HCC tissue bank, it was further confirmed that the levels of RBM39 were obviously higher in tumors than in adjacent noncancerous tissues (Supplementary Fig. S4B–D). Data from TCGA also revealed that HCC patients with high RBM39 expression had a shorter OS (Supplementary Fig. S4E). Interestingly, knockdown or overexpression of LINC01977 did not change the RNA or protein expression levels of RBM39 (Supplementary Fig. S5A–C). RBM39 was further analyzed in mouse xenograft tumor tissues and cell lines using IF staining. The data indicated that knockdown or overexpression of LINC01977 did not alter the localization and expression levels of RBM39 (Fig. 4L, M and Supplementary Fig. S5D, E). Furthermore, overexpression of RBM39 reversed the inhibition of cell proliferation and metastasis in MHCC97H cells with LINC01977 knockdown (Fig. 4N, O). Additionally, knockdown of RBM39 suppressed LINC01977-overexpression induced cell proliferation and metastasis in SMMC7721 cells (Supplementary Fig. S5F, G). Collectively, these results suggest that LINC01977 interacts with RBM39 and thus promotes the progression of HCC.

### LINC01977 is closely associated with the Notch signaling pathway and prevents Notch2 ubiquitination

**Fig. 5:**
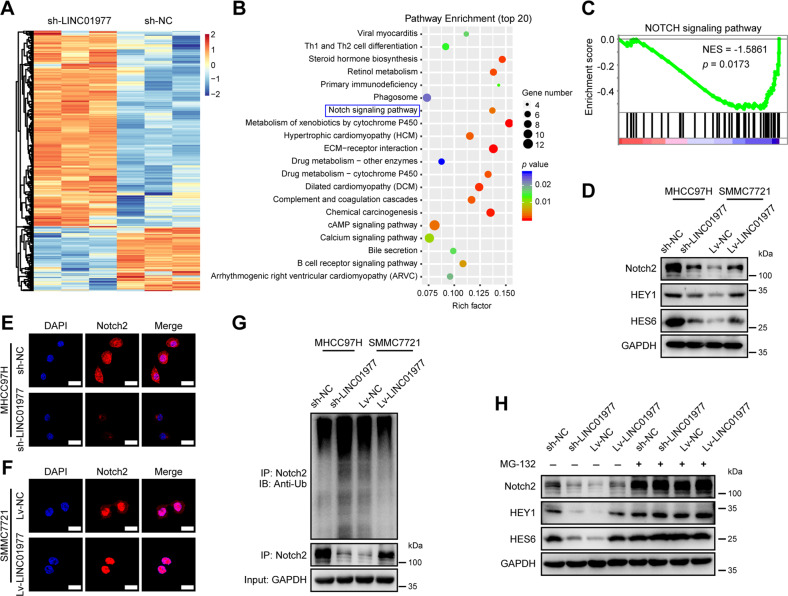
LINC01977 is closely associated with the Notch signaling pathway and prevents Notch2 ubiquitination. **A** Heatmap showing the differentially expressed genes in HCC cells with LINC01977 knockdown. **B** Top 20 KEGG pathways in HCC cells with LINC01977 knockdown. **C** GSEA plots of Notch signaling pathway-related signatures in HCC cells with LINC01977 knockdown. The nominal *P* value shows the statistical significance of the enrichment score analyzed by GSEA; NES, normalized enrichment score. **D** Immunoblot images of Notch2, HEY1 and HES6 in different HCC cells with the knockdown or overexpression of LINC01977. **E**, **F** IF staining of Notch2 in different HCC cells with LINC01977 knockdown or overexpression (scale bars = 25 μm). **G** Immunoprecipitation with Notch2 antibody and cell lysates and immunoblot images of ubiquitination. **H** Immunoblot images of Notch2, HEY1 and HES6 in different HCC cells with the knockdown or overexpression of LINC01977 cultured with or without MG132 treatment.

To further investigate the downstream mechanisms of LINC01977 in HCC, RNA-sequencing assay was carried out in LINC01977 knockdown and control cells. The results showed that knockdown of LINC01977 significantly resulted in upregulation of 478 genes and downregulation of 154 genes (fold change ≥2, *P* ≤ 0.05) (Fig. 5A). The top 20 differential signaling pathways were evaluated by Kyoto Encyclopedia of Genes and Genomes (KEGG), and the Notch signaling pathway was significantly enriched (Fig. 5B). Gene set enrichment analysis (GSEA) also demonstrated significant differences in Notch signaling pathways (Fig. 5C). Notch2, as a key gene in this signaling pathway, was identified. Western blotting suggested that knockdown of LINC01977 reduced Notch2 expression, whereas overexpression of LINC01977 increased Notch2 expression (Fig. 5D). The target genes of Notch2, such as *HEY1* and *HES6*, were further verified, and the data showed that LINC01977 positively regulated the expression of HEY1 and HES6 (Fig. 5D). Surprisingly, IF assays found that knockdown of LINC01977 led to the entry of Notch2 from the nucleus into the cytoplasm and reduced Notch2 levels (Fig. 5E). Overexpression of LINC01977 caused Notch2 to enter the nucleus from the cytoplasm and increased Notch2 levels (Fig. 5F). To elucidate this finding, the ubiquitin-proteasome degradation pathway was investigated. We performed an immunoprecipitation (IP) and western blotting assays. The data revealed that knockdown of LINC01977 enhanced the ubiquitination level of Notch2 protein in MHCC97H cells, while overexpression of LINC01977 inhibited the ubiquitination level of Notch2 protein in SMMC7721 cells (Fig. 5G). MG132, a proteasome inhibitor, was used, and the data revealed that MG132 abolished the regulatory effect of LINC01977 on Notch2 and its target genes (Fig. 5H). Taken together, these data indicate that LINC01977 is closely associated with the Notch signaling pathway and regulates Notch2 in a ubiquitination-dependent degradation pathway.

### Notch2 ubiquitination is mediated by RBM39, and Notch2 is involved in LINC01977-induced progression of HCC

**Fig. 6:**
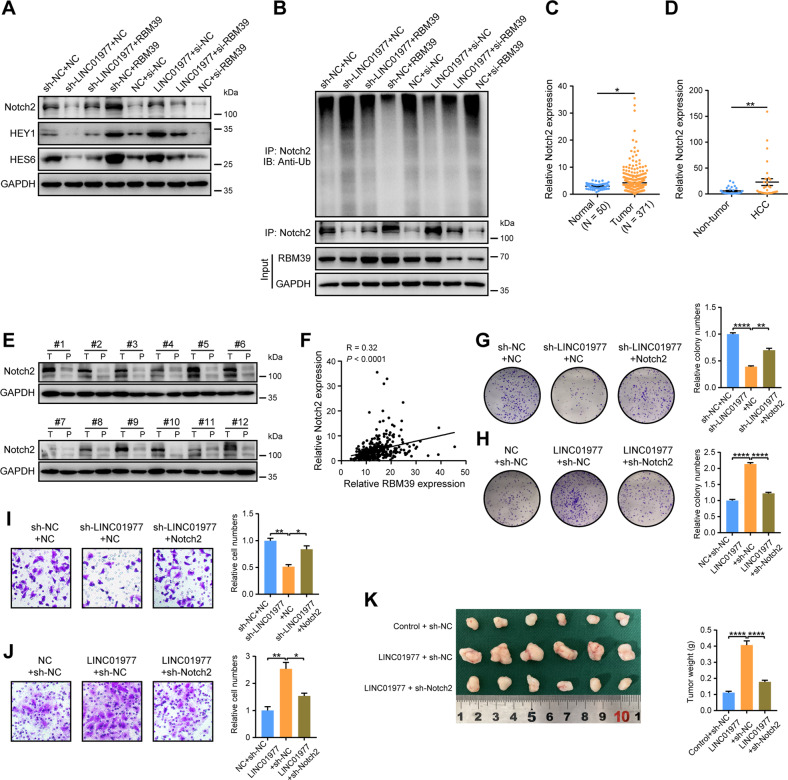
Notch2 ubiquitination is mediated by RBM39, and Notch2 is involved in LINC01977-induced progression of HCC. **A** Immunoblot images showing the expression levels of Notch2, HEY1 and HES6 after LINC01977 and/or RBM39 overexpression or knockdown in different HCC cells. **B** Immunoprecipitation with Notch2 antibody and different cell lysates after LINC01977 and/or RBM39 overexpression or knockdown and immunoblot images of ubiquitination. **C** The RNA levels of Notch2 in HCC (*n* = 371) and normal tissues (*n* = 50) from the TCGA database. **D** The RNA levels of Notch2 in HCC and paired nontumor tissues (*n* = 30). **E** The protein levels of Notch2 in HCC and paired nontumor tissues (*n* = 12). **F** Pearson correlation analysis between RBM39 expression and Notch2 expression. **G** Overexpression of Notch2 rescued the colony formation ability of MHCC97H cells with LINC01977 knockdown. **H** Knockdown of Notch2 rescued the colony formation ability of SMMC7721 cells overexpressing LINC01977. **I** Overexpression of Notch2 rescued the invasion ability of MHCC97H cells with LINC01977 knockdown. **J** Knockdown of Notch2 rescued the invasion ability of SMMC7721 cells overexpressing LINC01977. **K** Knockdown of Notch2 significantly suppressed LINC01977-induced tumor growth in nude mice (*n* = 6 per group). The tumor weights are shown. ^*^P < 0.05; ^**^P < 0.01; ^****^P < 0.0001.

To clarify whether RBM39 mediates the expression and ubiquitination of Notch2, rescue assays were performed. First, overexpression of RBM39 reversed the reduction in Notch2 protein levels in MHCC97H cells caused by LINC01977 knockdown (Fig. 6A). Knockdown of RBM39 suppressed the upregulation of Notch2 protein levels in SMMC7721 cells induced by LINC01977 overexpression (Fig. 6A). Additionally, RBM39 mediated the regulatory effect of LINC01977 on Notch2 target genes such as *HEY1* and *HES6* (Fig. 6A). In addition, IP and ubiquitination analyses showed that overexpression of RBM39 inhibited Notch2 ubiquitination in MHCC97H cells induced by LINC01977 knockdown, whereas knockdown of RBM39 increased Notch2 ubiquitination in SMMC7721 cells with LINC01977 overexpression (Fig. 6B).

Furthermore, according to TCGA data, Notch2 was obviously higher in HCC tumor tissues (Fig. 6C). Additionally, high expression of Notch2 in tumors was also confirmed in our HCC tissue bank, both at RNA and protein levels (Fig. 6D, E). Pearson correlation analysis showed that RBM39 was positively correlated with Notch2 from the TCGA data (Fig. 6F). To elucidate whether Notch2 mediates the oncogenic effects of LINC01977, we performed related rescue experiments. Overexpression of Notch2 reversed the inhibition of cell proliferation and invasion in MHCC97H cells by LINC01977 knockdown, whereas knockdown of Notch2 suppressed LINC01977-overexpression induced cell proliferation and metastasis in SMMC7721 cells (Fig. 6G–J). Consistently, tumor xenograft models uncovered that knockdown of Notch2 obviously inhibited LINC01977-induced tumor growth (Fig. 6K). Therefore, these data indicate that Notch2 ubiquitination is mediated by RBM39 and that Notch2 mediates the oncogenic effects of LINC01977 in HCC.

### IGF2BP2 stabilizes LINC01977 in an m^6^A-dependent manner

**Fig. 7:**
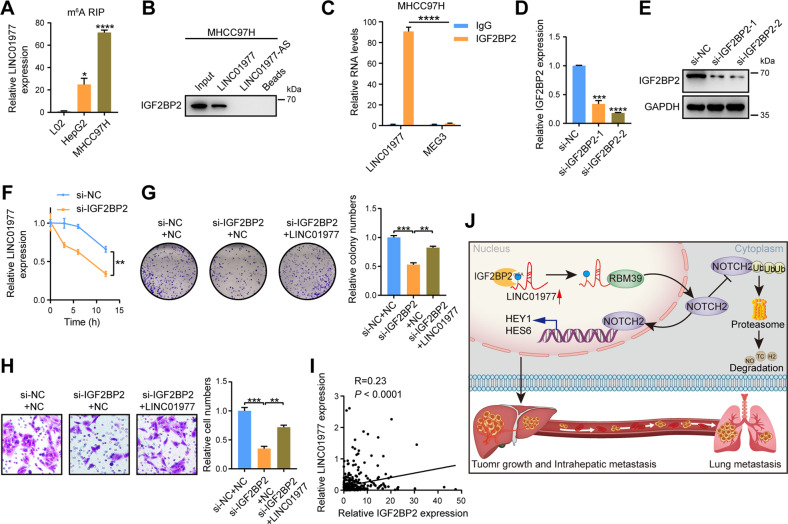
IGF2BP2 stabilizes LINC01977 in an m^6^A-dependent manner. **A** The m^6^A RIP–qPCR analysis of LINC01977 in HepG2 and MHCC97H cells. **B** Immunoblot images of the specific interaction between LINC01977 and IGF2BP2. **C** RIP assays showing the interaction between IGF2BP2 and LINC01977 in MHCC97H cells. MEG3 serves as an abundant nuclear lncRNA control. **D** The knockdown efficiencies of IGF2BP2 at the RNA level in MHCC97H cells. **E** The knockdown efficiencies of IGF2BP2 at the protein level in MHCC97H cells. **F** MHCC97H cells with IGF2BP2 knockdown or control cells were treated with actinomycin D for the indicated times, and LINC01977 expression was measured. **G** Overexpression of LINC01977 rescued the colony formation ability of MHCC97H cells with IGF2BP2 knockdown. **H** Overexpression of LINC01977 rescued the invasion ability of MHCC97H cells with IGF2BP2 knockdown. **I** Pearson correlation analysis between IGF2BP2 expression and LINC01977 expression. **J** A schematic abstract of the IGF2BP2-LINC01977-RBM39-Notch2 axis promoting HCC progression. ^*^P < 0.05; ^**^P < 0.01; ^***^P < 0.001; ^****^P < 0.0001.

An increasing number of researches have indicated that m^6^A modification exerts an important role in the stability of lncRNAs. To confirm the existence of m^6^A modifications in LINC01977, RIP was carried out. The data demonstrated that m^6^A was clearly enriched in LINC01977 from MHCC97H and HepG2 cells (Fig. 7A). Interestingly, we reanalyzed the MS of the pulldown assay for LINC01977 and identified IGF2BP2 among these pulldown proteins. Western blotting and RIP assays together indicated that IGF2BP2 and LINC01977 bound to each other (Fig. 7B, C). However, knockdown or overexpression of LINC01977 did not affect the RNA and protein expression levels of IGF2BP2 in HCC cells (Supplementary Fig. S6A–C). IGF2BP2 is a known m^6^A reader that recognizes m^6^A modification sites (GGACs). LINC01977 has this modification site, indicating that IGF2BP2 may have a stabilizing effect on LINC01977 in HCC cells. In view of this, we first verified the knockdown efficiency of IGF2BP2 in MHCC97H cells (Fig. 7D, E). Then, stability assays using actinomycin D were performed. The results showed that inhibition of IGF2BP2 resulted in a rapid reduction in the half-life of LINC01977, indicating that IGF2BP2 increased the stability of LINC01977 in MHCC97H cells (Fig. 7F). Moreover, overexpression of LINC01977 reversed the inhibition of cell proliferation and metastasis in MHCC97H cells by IGF2BP2 knockdown (Fig. 7G, H). Pearson correlation analysis further showed that IGF2BP2 was positively correlated with LINC01977 (Fig. 7I). In conclusion, these results suggest that LINC01977 is stabilized by IGF2BP2 and thus promotes the development of HCC via the IGF2BP2-LINC01977-RBM39-Notch2 axis (Fig. 7J).

## Discussion

HCC serves as one of the most common gastrointestinal malignancies, and the underlying pathological mechanisms of HCC occurrence and metastasis yet have much room for exploration [2, 3]. In recent years, lncRNAs have been found to regulate the development of various tumors by interacting with proteins or nucleic acids [17, 23, 24]. Here, we discover a novel CT-lncRNA, LINC01977, through the TCGA and GTEx databases. At the tissue level, LINC01977 is exclusively expressed in testes and highly expressed in HCC. At the cellular level, LINC01977 is highly expressed in common hepatoma cell lines compared with normal cells. Prognostic analysis shows that patients with high LINC01977 expression have a lower OS. Also, LINC01977 is a prognostic factor for OS in patients with HCC. Functional experiments demonstrate that LINC01977 promotes HCC progression. Mechanistically, LINC01977 binds to RBM39, promotes the further entry of Notch2 into the nucleus, reduces the ubiquitination of Notch2, and increases the stability of Notch2. Notch2 mediates the tumor-promoting effect of LINC01977 in HCC. Furthermore, IGF2BP2, as an m^6^A reader, increases the stability of LINC01977 in HCC cells. Therefore, these data reveal that LINC01977 exerts a key role in the progression of HCC.

Since lncRNAs generally function in association with proteins, we perform RNA pulldown analysis and identify RBM39 as the protein bound to LINC01977. RBPs are critical regulators of gene expression, and changes in these proteins are involved in human diseases, including various cancers [19, 25–28]. It has been previously reported that the lncRNA DARS-AS1 bound to RBM39 and promoted the malignant progression of myeloma [29]. DARS-AS1 bound to RBM39, hindered the interaction between RBM39 and RNF147, and prevented ubiquitination and degradation of RBM39. Furthermore, RBM39 was upregulated in HCC, which was associated with the appearance of microvessels [30]. Our findings confirm that RBM39 is an oncogene, and that LINC01977 and RBM39 are colocalized in the nucleus. Rescue experiments also confirm that LINC01977 interacts with RBM39 to promote the progression of HCC. However, alterations in LINC01977 expression levels do not affect RBM39 expression levels or RBM39 localization. Thus, studies are further required to estimate whether LINC01977 affects other unknown aspects such as posttranslational modification of RBM39.

Increasing researches have reported that the Notch signaling pathway is closely related to the progression of cancer, including HCC [31–33]. To further investigate the mechanisms associated with LINC01977, we perform RNA sequencing to identify pathways affected by LINC01977, and the Notch signaling pathway is significantly enriched. Studies have reported that Notch2 was a key molecule in the Notch signaling pathway and that Notch2 promoted the malignant progression of HCC [32, 33]. Furthermore, lncAKHE interacted with YEATS4 to enhance the activation of NOTCH2, thus promoting HCC development [34]. In our study, LINC01977 affects Notch2 through the proteasome–ubiquitination pathway. First, knockdown or overexpression of LINC01977 inhibits or increases the expression of Notch2 protein. LINC01977 increases the nuclear localization of Notch2 and reduces its ubiquitination and degradation in the cytoplasm. Additionally, RBM39 overexpression or knockdown mediates ubiquitination and degradation of Notch2 by LINC01977. In addition, corresponding rescue experiments are performed. The data confirm that knockdown of Notch2 inhibits LINC01977-induced HCC growth and metastasis, while the elevation of Notch2 reverses the inhibition of cell proliferation and metastasis by LINC01977 knockdown. These results suggest that LINC01977 binds to RBM39, which in turn affects Notch signaling through the proteasome–ubiquitination pathway.

It has been reported that RNA has many modifications including m^6^A modification [35–38]. The m^6^A methylation process is dynamically determined by methyltransferases (writers), demethylases (erasers), and effector proteins (readers) [39]. Among them, effector proteins include YTHDC1, IGF2BP1, IGF2BP2 and IGF2BP3, which could identify m^6^A-modified transcripts. They play important roles in various aspects such as RNA stability, translation efficiency, RNA splicing, and RNA export [18, 40–42]. In our study, the m^6^A enrichment of LINC01977 is very high in HCC cell lines. Analysis of the MS results and experiments both reveals that LINC01977 binds to IGF2BP2. The stability of LINC01977 is significantly decreased after knockdown of IGF2BP2. Therefore, IGF2BP2 acts as an m^6^A reader and enhances the stability of LINC01977.

Overall, our study demonstrates LINC01977 as a CT-lncRNA. LINC01977 is exclusively expressed in testes and highly expressed in HCC, which promotes HCC progression. Mechanistic studies reveal that IGF2BP2 enhances the stability of LINC01977, resulting in its high level. Meanwhile, LINC01977 binds to RBM39, promotes the further entry of Notch2 into the nucleus, and prevents the ubiquitination and degradation of Notch2. Therefore, LINC01977 exerts a key role in the occurrence and development of HCC through the IGF2BP2-LINC01977-RBM39-Notch2 axis, which suggests that LINC01977 could be a promising target for patients with HCC.

## Materials and methods

### Clinical samples

Fresh tumor tissues, adjacent noncancerous tissues and normal tissues were all acquired from the Affiliated Drum Tower Hospital, Medical School of Nanjing University. None of the HCC patients received chemotherapy or radiotherapy before resection, and all participating patients signed the relevant informed consent before surgery. All tissues after surgical resection were immediately put into liquid nitrogen for rapid freezing. Approval for this study was obtained from the Institutional Ethics Committee of the Affiliated Drum Tower Hospital, Medical School of Nanjing University. All researches were in compliance with government policies and the Declaration of Helsinki.

### Cell culture

The human cell lines (L02, Hep3B, SMMC7721, Huh7, MHCC97H and MHCC97L) were purchased from the cell bank of the Chinese Science Academy. HepG2 and HEK293T cells were purchased from the American Type Culture Collection (ATCC). Cells were cultured in Dulbecco’s modified Eagle’s medium (Invitrogen Life Technologies) supplemented with 10% fetal bovine serum and incubated in a humidified incubator containing 5% CO_2_ at 37 °C. All cell lines were routinely monitored for mycoplasma and were negative.

### Lentiviral infection

Short hairpin RNA (shRNA) targeting LINC01977 was purchased from GenePharma. The lentivirus overexpressing LINC01977 was purchased from Vigene. Briefly, lentiviral packaging required cotransfection of target and packaging plasmids (psPAX2 and pMD2.G) into HEK293T cells using transfection reagents. After 48 h, the supernatant containing lentiviral particles was collected and filtered. Cells were infected with lentiviruses and screened for positively transfected cells with puromycin. Finally, the efficiency of transfection was verified by qRT-PCR. All sequences are listed in Supplementary Table 2.

### Proliferation assays

Colony formation assays were performed by seeding cells in six-well plates at 400 cells per well and placing them in an incubator for 10 days. Then, the cells were stained with crystal violet (Beyotime) and counted. Cell viability was assessed using the EdU assay (RiboBio). Soft agar colony formation assays required different concentrations of agar solutions followed by HCC cell suspension preparation and incubator placement for 14 days. For the HCC organoid assay, HCC organoids were first established with human HCC fresh tissues, and then the organoids were transfected with LINC01977-overexpressing lentivirus and control lentivirus. After 14 days, images of HCC organoids were obtained by light microscopy.

### Angiogenesis assay

HCC cells were first added to six-well plates and placed in an incubator. Then, the supernatant was collected and filtered through a 0.22-micron filter. Filtered supernatants were used to resuspend HUVECs, and the cells were seeded in 50 μl Matrigel-coated 96-well plates. After 6 h, the tubular structures were observed with a microscope and photographed for analysis.

### Migration and invasion assays

For the Transwell assay, the upper chamber was seeded with HCC cells in serum-free medium, with or without Matrigel (BD Biosciences). The lower chamber was filled with 700 μl of medium containing 10% FBS, and the cells were placed in an incubator for 48 h. Then, the cells were stained with crystal violet (Beyotime) and counted.

### RNA isolation and quantitative real-time PCR (qRT-PCR)

TRIzol reagent (Invitrogen Life Technologies) was utilized to obtain RNA from tissues and cells. cDNA was derived from one microgram of RNA using HiScript Q RT SuperMix (Vazyme). qRT-PCR was carried out with the SYBR Green PCR Kit (Vazyme) and detected by the Applied Biosystems 7900HT Sequence Detection System (Applied Biosystems). Primers are listed in Supplementary Table 3.

### Western blotting

Cell or tissue proteins were extracted using RIPA lysis buffer with protease and phosphatase inhibitors. The protein concentration was determined by a BCA Protein Assay Kit (Beyotime). Approximately 30 μg of protein was loaded per lane. The proteins were separated by 10% SDS-PAGE and transferred to a 0.45 μm polyvinylidene fluoride (PVDF) membrane. The primary antibody was incubated with the membrane overnight at 4 °C. The membranes were washed with TBST, incubated with the corresponding secondary antibodies for 2 h at room temperature, and then washed with TBST. Finally, the target protein bands were detected using ECL detection reagent. The antibodies used are listed in Supplementary Table 4.

### mRNA stability assay

Actinomycin D (MCE) was added to cells to restrain de novo RNA synthesis. At different time points, cells were harvested with TRIzol reagent. Finally, total cellular RNA was acquired and qRT-PCR was carried out for further analysis.

### Immunohistochemistry (IHC)

Eight-micrometer-thick paraffin-embedded tissue was transferred to a glass slide. The slides were then deparaffinized and hydrated. Tris-EDTA pH 9.0 was utilized for antigen retrieval. Intrinsic peroxidase activity was inactivated and serum blocking was performed at room temperature. Ki67 antibody (1:200; Abcam) and RBM39 antibody (1:200; Proteintech) were added to the tissues and incubated for 13 h at 4 °C. Next, the slides were washed and biotinylated secondary antibody was added to the slides for incubation. Finally, the tissues were colored with DAB solution, dehydrated with ethanol and mounted. Light microscopy was used to take photos and analyze the samples.

### Immunofluorescence (IF)

For tissue immunofluorescence, slides were deparaffinized and hydrated. Tris-EDTA pH 9.0 was utilized for antigen retrieval. Intrinsic peroxidase activity was inactivated, and serum blocking was performed at room temperature. CD31, E-cadherin, Vimentin and RBM39 antibodies were added to the tissues and incubated for 13 h at 4 °C. Antibody concentrations were all diluted 1:200. Next, the slides were washed and Alexa Fluor-labeled secondary antibodies (Abcam) were added to the slides for incubation. Finally, the tissues were stained with DAPI solution for nuclei and photographed with a confocal microscope (Leica).

For cellular immunofluorescence, cells were first fixed with 4% paraformaldehyde. Then, serum blocking was performed at room temperature. E-cadherin, Vimentin and RBM39 antibodies were added to the cells and incubated for 13 h at 4 °C. Antibody concentrations were all diluted 1:200. Next, the cells were washed and Alexa Fluor-labeled secondary antibodies (Abcam) were added to the cells. Finally, the cells were stained with DAPI solution for nuclei and photographed using a confocal microscope (Leica).

### Subcellular RNA fractionation

Cytoplasmic and nuclear RNAs were separated and purified from MHCC97H and HepG2 cells using the Cytoplasmic and Nuclear RNA Purification Kit (Norgen). The RNAs were then reverse transcribed into cDNAs, and qRT-PCR was utilized to determine the expression levels of LINC01977, GAPDH and U2 in the cytoplasm and nucleus. GAPDH and U2 were used as cytoplasmic and nuclear controls, respectively.

### Fluorescence in situ hybridization (FISH)

MHCC97H and HepG2 cells were subjected to FISH using an RNA FISH kit (RiboBio). The target probe LINC01977 and control probes (18 S and U6) were supplied by RiboBio and labeled with Cy3. Firstly, cells were seeded in confocal dishes. When the cells are completely attached, a series of subsequent operations such as fixation and permeabilization were carried out. The prehybridization solution was used for blocking. The corresponding probes were utilized for overnight incubation to allow the probes to bind to the targets. Finally, the cells were stained with DAPI solution for nuclei and photographed using a confocal microscope (Leica).

### RNA pulldown assay

The full-length LINC01977 plasmid was cloned into the pGEM-3Z vector. First, linear plasmids were cut enzymatically and transcribed into biotinylated RNA in vitro using T7 and Sp6 RNA polymerases. Magnetic beads, biotinylated RNA, and MHCC97H cell lysates were coincubated at 4 °C using a Pierce Magnetic RNA-Protein Pull-Down kit (Thermo Fisher Scientific). Finally, proteins were eluted and subjected to mass spectrometry and western blotting analyses.

### RNA immunoprecipitation (RIP)

First, sufficient cell lysate was obtained from MHCC97H cells. The beads were then eluted and bound by incubation with the corresponding antibodies IgG, RALY (Novus), DHX9 (Novus), RBM39 (Proteintech), m^6^A (Abcam) and IGF2BP2 (Proteintech). Purified RNA was obtained by incubating the lysate with the magnetic bead-antibody complex overnight at 4 °C. Finally, qRT-PCR was utilized to verify the expression of LINC01977, MEG3 and IgG.

### RNA sequencing

First, total RNA was acquired from LINC01977 knockdown cells and control cells with TRIzol reagent. The RNA samples were then sequenced using the Illumina HiSeq 4000 platform (Illumina) to generate raw data (LC-Bio). The genes with significant differential expression were chosen based on fold change ≥2.0 and *P* ≤ 0.05. Volcano map, heatmap, KEGG and GSEA pathway analyses were explored. Each group had three samples. Raw data were deposited in the Gene Expression Omnibus (GEO) database (GSE227449).

### Animal studies

BALB/c male nude mice (6–8 weeks old) were obtained from GemPharmatech Co., Ltd and maintained in specific pathogen-free facilities. Mice were housed at 20 ± 2 °C under a 12-h light/12-h dark photoperiod with lights on at 7:00. All studies involving animals were approved by the Animal Care and Use Committee of the Affiliated Drum Tower Hospital, Medical School of Nanjing University. All researches were in compliance with the Guide for the Care and Use of Laboratory Animals.

For the subcutaneous tumor model, stable HCC cell lines were first constructed and inoculated subcutaneously into nude mice. The number of cells per mouse was 5 million, with six mice per group. After 4 weeks, tumors were analyzed by bioluminescence imaging. Subcutaneous tumors of nude mice were then removed, tumor volumes were measured, and tumor weights were weighed. Finally, tumor tissue was prepared into slides for HE, IHC and IF analyses.

For the surgical orthotopic implantation, subcutaneous xenograft tumors were first obtained and then divided into small tissues of ~1 mm^3^. Nude mice were anesthetized, the liver was exposed with scissors and forceps, and a piece of tumor was placed in the liver. After 3 weeks, liver metastases were evaluated by bioluminescence imaging. Livers were then removed and used for HE analysis.

For the lung metastasis model, stable HCC cells were first constructed and then injected into nude mice via the tail vein. The number of cells in each nude mouse was 1 million, and there were six in each group. After 6 weeks, lung metastases were evaluated by bioluminescence imaging. Lungs were then removed and used for HE analysis.

For survival analysis, stable HCC cells were constructed first, and then control and LINC01977-overexpressing cells were infused into nude mice. The number of cells in each nude mouse was 1 million, and there were ten in each group. The total observation period was 80 days. Based on the survival time, a survival curve was constructed.

Sample sizes were generally chosen based on preliminary data indicating the differences between groups. No statistical method was used to predetermine sample size. Mice were randomly assigned to the groups before treatments, and the investigators were blinded to the allocation of mice into different treatment groups.

### Statistical analysis

Data are shown as the mean ± s.e.m. Comparisons between two groups were performed using a two-tailed unpaired or paired Student’s *t* test. The chi-square test assessed the correlation between LINC01977 expression and clinicopathological features. Kaplan–Meier curves described the survival function and the log-rank test evaluated the significant differences between different groups. HR and 95% CIs were assessed by univariate and multivariate Cox regression analyses. Each experiment was performed at least three times. Statistical analysis of the data was conducted with SPSS and GraphPad softwares. All *P* values are *P < 0.05, **P < 0.01, ***P < 0.001 and ****P < 0.0001.

## Supplementary information

supplementary data

Original Data File

## Data Availability

The data that support the findings of this study are available from the corresponding author upon reasonable request.
