# Supplementary material for: The cancer-testis lncRNA LINC01977 promotes HCC progression by interacting with RBM39 to prevent Notch2 ubiquitination
Source: Cell Death Discov. 2023 May 18;9:169. doi: 10.1038/s41420-023-01459-1 (PMC10192213; doi:10.1038/s41420-023-01459-1)
Supplement: Supplementary file 1 — supplementary data [file 41420_2023_1459_MOESM1_ESM.docx]

**Supplementary Figures**

**
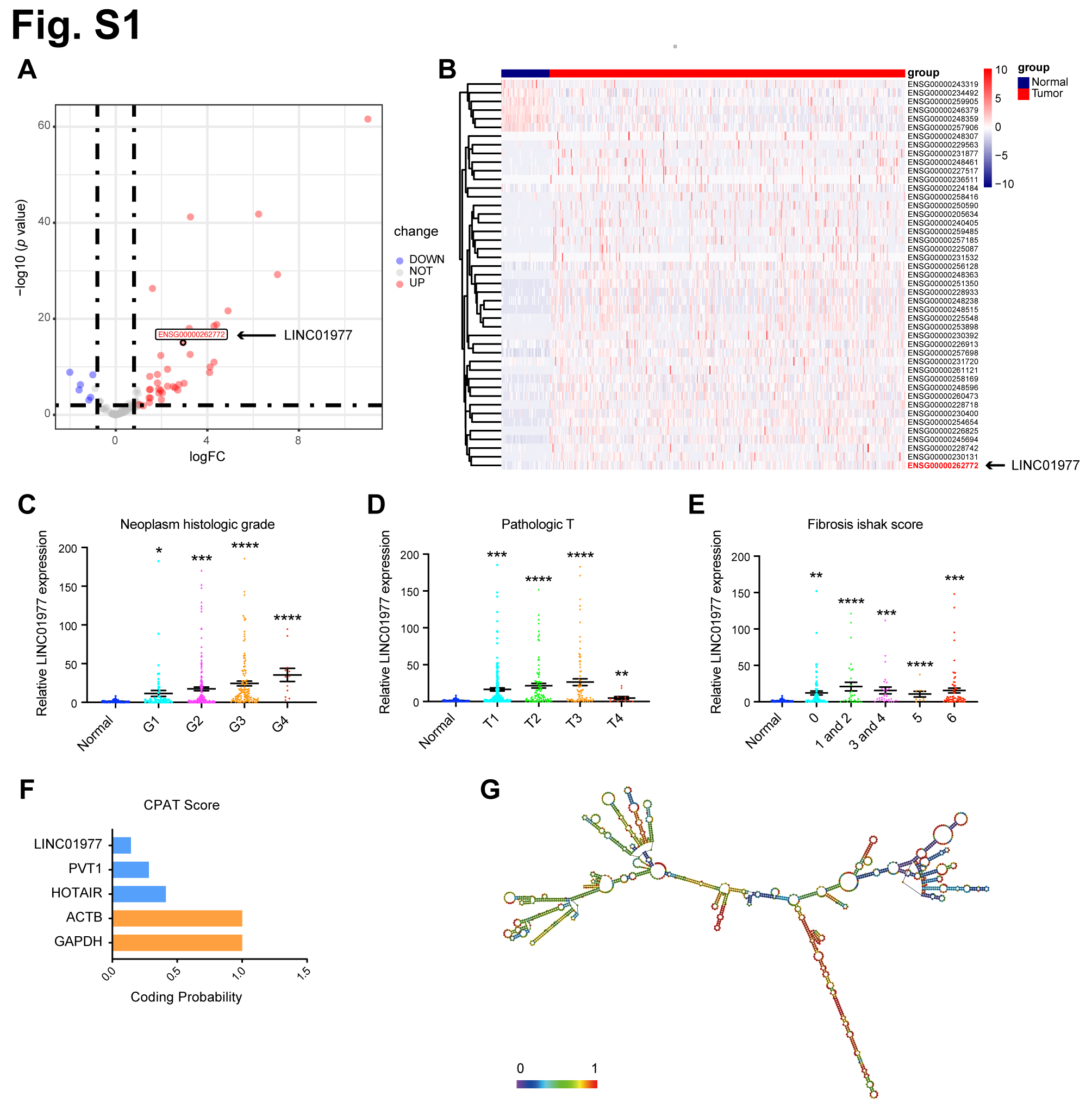
**

**Figure S1. The characteristics of LINC01977.**

(A) The expression levels of 171 testis-specific non-coding RNAs in HCC from the TCGA database using the volcano plot.

(B) The expression levels of testis-specific non-coding RNAs in HCC from the TCGA database using the heat map (adjP < 0.05, |fold change| > 2).

(C) The expression levels of LINC01977 in HCC from the TCGA database based on neoplasm histologic grade (G1: well differentiated, G2: moderately differentiated, G3: poorly differentiated, G4: undifferentiated/anaplastic).

(D) The expression levels of LINC01977 in HCC from the TCGA database based on pathologic T (T1: solitary tumor without vascular invasion, T2: solitary tumor with vascular invasion or multiple tumors, none more than 5 cm in greatest dimension, T3: multiple tumors any more than 5 cm or tumor involving a major branch of the portal or hepatic vein, T4: tumor(s) with direct invasion of adjacent organs other then the gallbladder or with perforation of visceral peritoneum).

(E) The expression levels of LINC01977 in HCC from the TCGA database based on fibrosis ishak score (0: No Fibrosis, 1 and 2: Portal Fibrosis, 3 and 4: Fibrous Speta, 5: Nodular Formation and Incomplete Cirrhosis, 6: Established Cirrhosis).

(F) The protein-coding potential of LINC01977 using the Coding Potential Assessment Tool (http://lilab.research.bcm.edu/cpat/). GAPDH and β-actin served as the positive controls for coding genes, and PVT1 and HOTAIR served as the positive controls for non-coding genes.

(G) The secondary structure of LINC01977 by the Vienna RNA Web Services (http://rna.tbi.univie.ac.at/).

**
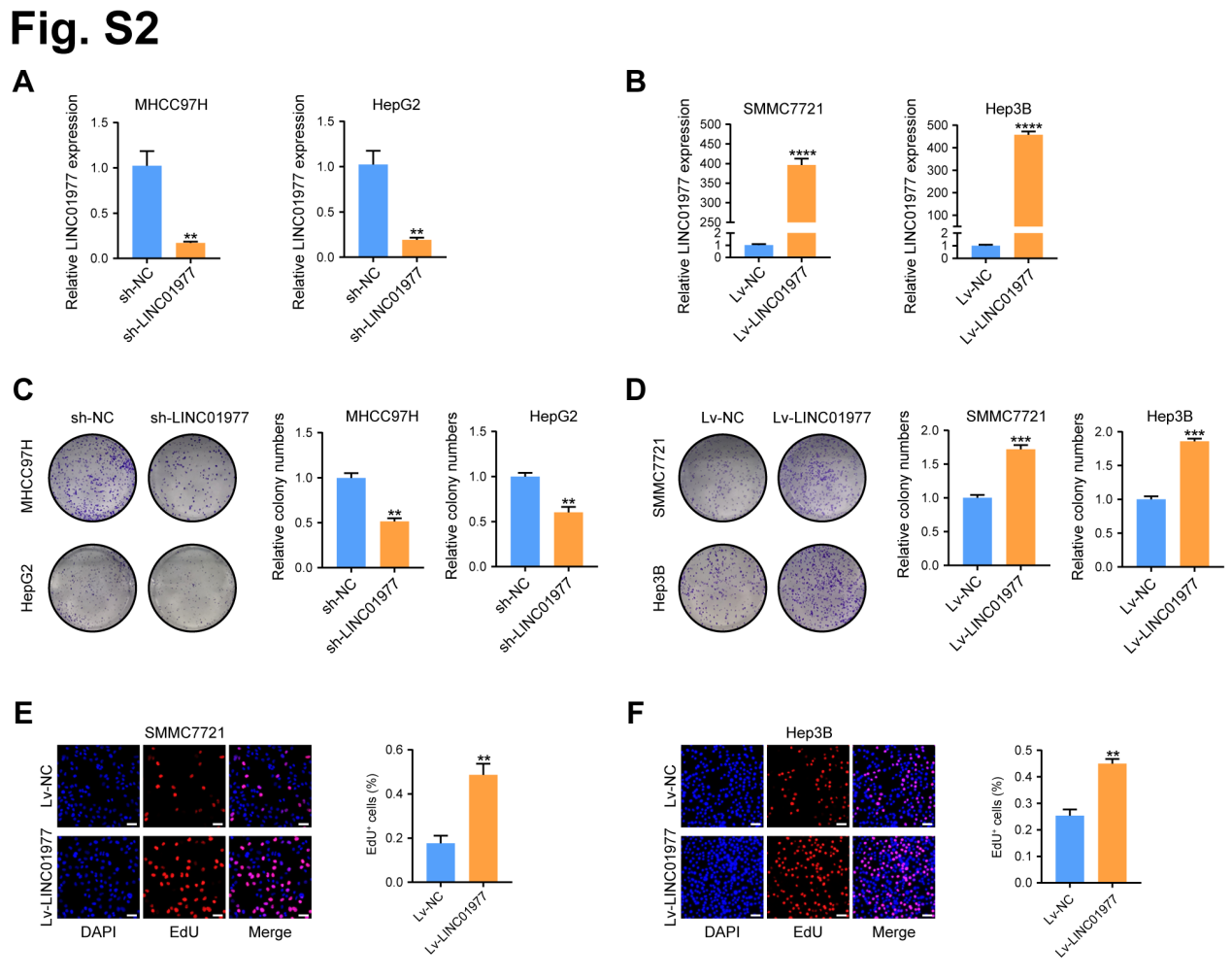
**

**Figure S2. The knockdown and overexpression efficiencies and cell proliferation assays in HCC cells.**

(A) The knockdown efficiencies of LINC01977 at the RNA level in different HCC cells.

(B) The overexpression efficiencies of LINC01977 at the RNA level in different HCC cells.

(C) Colony formation assays in MHCC97H and HepG2 cells with LINC01977 knockdown.

(D) Colony formation assays in SMMC7721 and Hep3B cells overexpressing LINC01977.

(E) EdU assays in SMMC7721 cells overexpressing LINC01977 (scale bars = 50 μm).

(F) EdU assays in Hep3B cells overexpressing LINC01977 (scale bars = 50 μm).

^**^*P* < 0.01; ^***^*P* < 0.001; ^****^*P* < 0.0001.

**
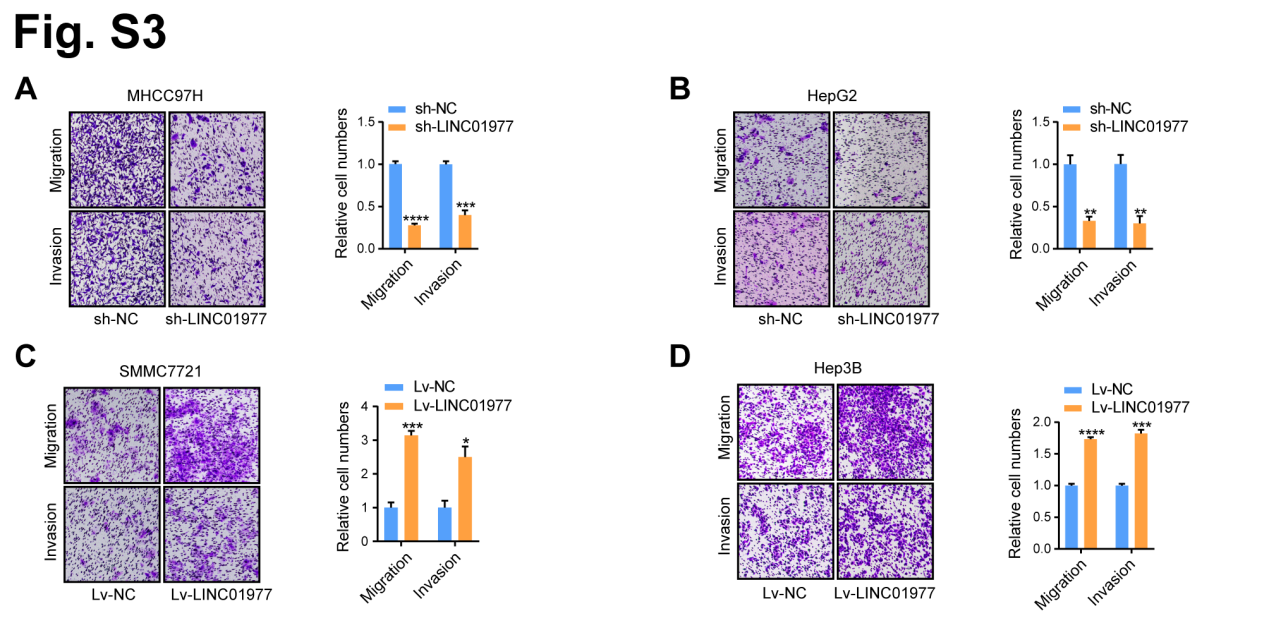
**

**Figure S3. LINC01977 promotes HCC migration and invasion in vitro.**

(A) Representative images of cell migration and invasion assays in MHCC97H cells with LINC01977 knockdown.

(B) Representative images of cell migration and invasion assays in HepG2 cells with LINC01977 knockdown.

(C) Representative images of cell migration and invasion assays in SMMC7721 cells overexpressing LINC01977.

(D) Representative images of cell migration and invasion assays in Hep3B cells overexpressing LINC01977.

^*^*P* < 0.05; ^**^*P* < 0.01; ^***^*P* < 0.001; ^****^*P* < 0.0001.

**
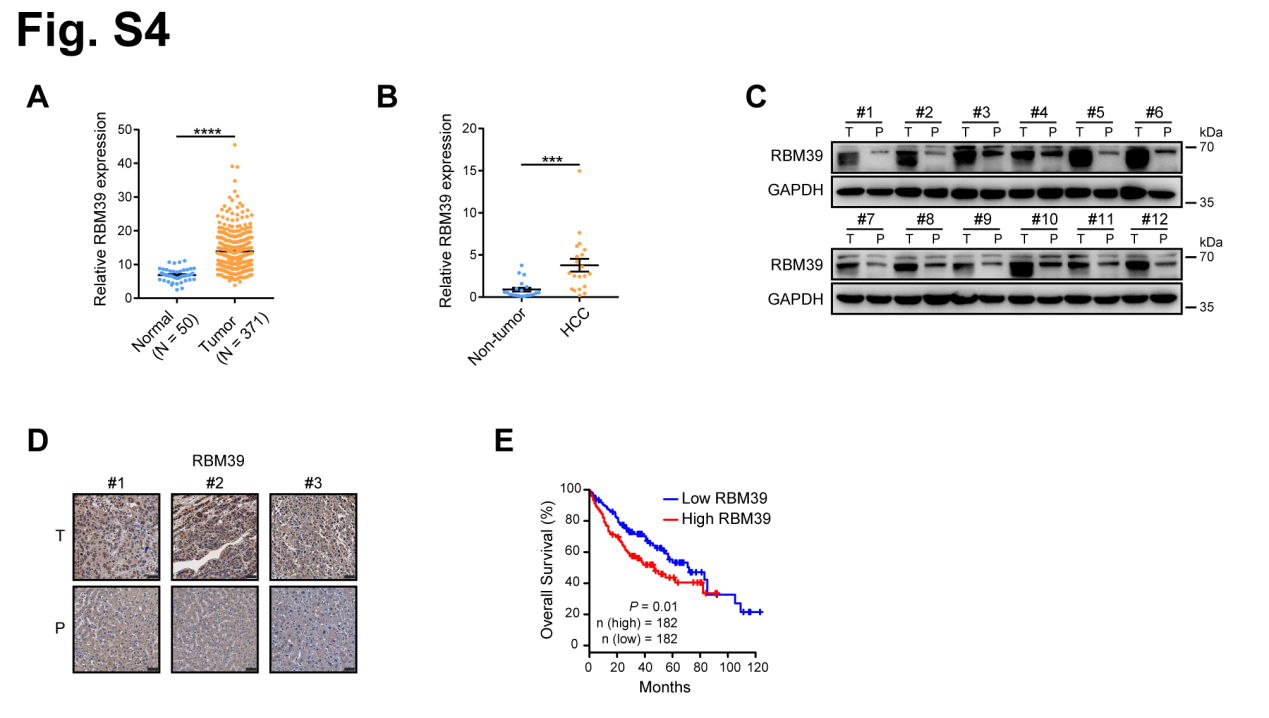
**

**Figure S4.** **RBM39 is highly expressed in HCC.**

(A) The RNA levels of RBM39 in HCC and nontumor tissues from the TCGA database.

(B) The RNA levels of LINC01977 in HCC and paired nontumor tissues (n = 20).

(C) The protein levels of RBM39 in HCC and paired nontumor tissues (n = 12).

(D) IHC staining of RBM39 in HCC and paired nontumor tissues (n = 3, scale bars = 50 μm).

(E) Kaplan–Meier survival curves of OS from TCGA database (n = 364, log-rank test).

^***^*P* < 0.001; ^****^*P* < 0.0001.

**
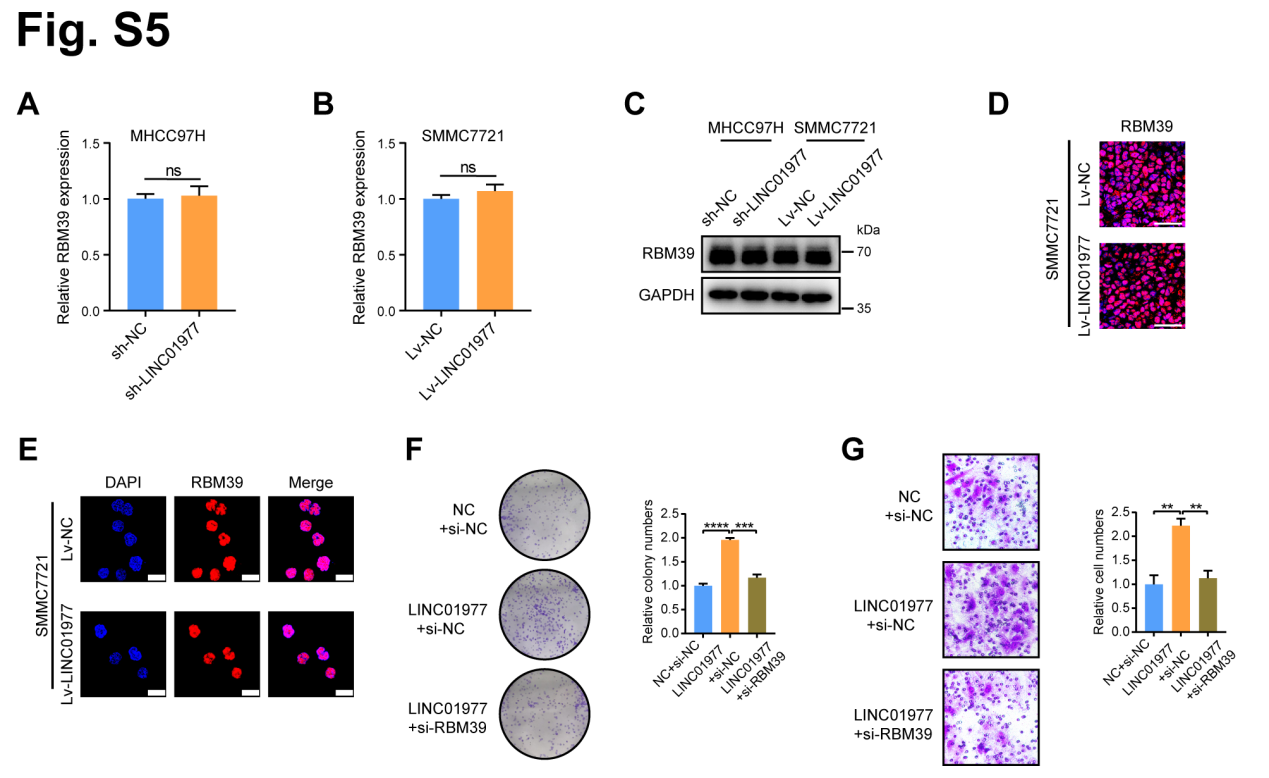
**

**Figure S5.** **LINC01977 has no effect on the expression and localization of RBM39.**

(A) The RNA levels of RBM39 in MHCC97H cells with LINC01977 knockdown.

(B) The RNA levels of RBM39 in SMMC7721 cells overexpressing LINC01977.

(C) The protein levels of RBM39 in different HCC cells with the knockdown or overexpression of LINC01977.

(D) IF staining of RBM39 in xenograft tumors with the overexpression of LINC01977 (scale bars = 50 μm).

(E) IF staining of RBM39 in SMMC7721 cells overexpressing LINC01977 (scale bars = 25 μm).

(F) Knockdown of RBM39 rescued the colony formation ability of SMMC7721 cells overexpressing LINC01977.

(G) Knockdown of RBM39 rescued the invasion ability of SMMC7721 cells overexpressing LINC01977.

^**^*P* < 0.01; ^***^*P* < 0.001; ^****^*P* < 0.0001.

**
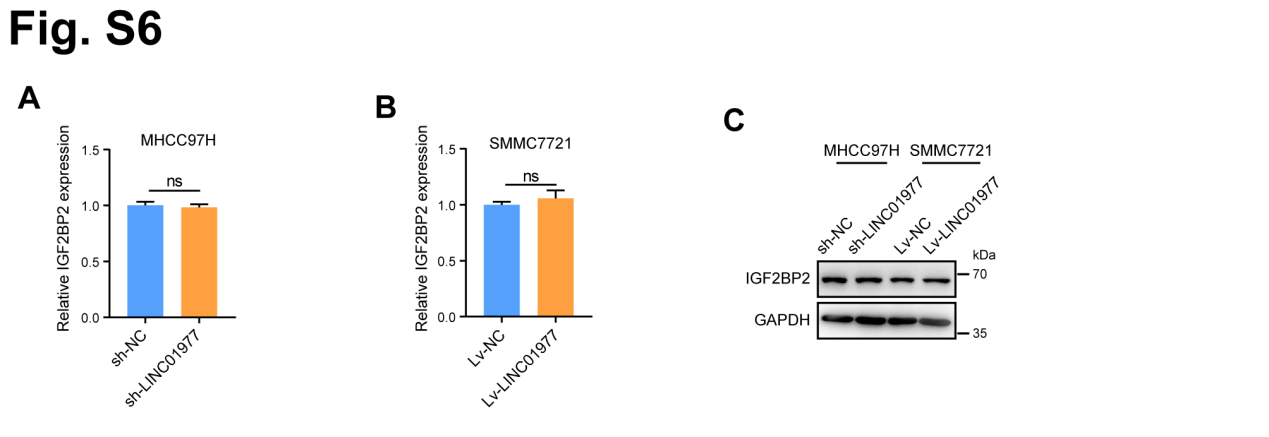
**

**Figure S6. The expression levels of IGF2BP2 in HCC cells with LINC01977 knockdown or overexpression.**

(A) The RNA levels of IGF2BP2 in MHCC97H cells with LINC01977 knockdown.

(B) The RNA levels of IGF2BP2 in SMMC7721 cells overexpressing LINC01977.

(C) The protein levels of IGF2BP2 in different HCC cells with the knockdown or overexpression of LINC01977.

**Supplementary Table 1: Correlation between LINC01977 expression and clinicopathological characteristics of HCC patients (*n* = 72**)

| **Characteristics** | Number of Patients | **LINC01977 low expression (<median)** | **LINC01977 high expression (≥median)** | ***P* value** |
| --- | --- | --- | --- | --- |
| All cases | 72 | 36 | 36 |  |
| Gender |  |  |  | 0.147 |
| Male | 57 | 26 | 31 |  |
| Female | 15 | 10 | 5 |  |
| Age(years) |  |  |  | 0.141 |
| ＜60 | 46 | 20 | 26 |  |
| ≥60 | 26 | 16 | 10 |  |
| Tumor size (cm) |  |  |  | **0.026** |
| ＜5 | 25 | 17 | 8 |  |
| ≥5 | 47 | 19 | 28 |  |
| Tumor number |  |  |  | **0.003** |
| Single | 53 | 32 | 21 |  |
| Multiple | 19 | 4 | 15 |  |
| HBV |  |  |  | 0.141 |
| Negative | 26 | 16 | 10 |  |
| Positive | 46 | 20 | 26 |  |
| AFP (ng/ml) |  |  |  | **0.004** |
| ＜200 | 51 | 31 | 20 |  |
| ≥200 | 21 | 5 | 16 |  |
| Cirrhosis |  |  |  | 0.157 |
| No | 38 | 22 | 16 |  |
| Yes | 34 | 14 | 20 |  |
| Differentiation grade |  |  |  | **0.000** |
| Ⅰ+Ⅱ | 34 | 27 | 7 |  |
| Ⅲ+Ⅳ | 38 | 9 | 29 |  |
| Vascular invasion  No  Yes | 44  28 | 28  8 | 16  20 | **0.004** |

The median expression level was used as the cutoff.

Data were analyzed by the chi-squared test. The *P* value in bold indicates statistical significance.

**Supplementary Table 2: The sequences of siRNAs and shRNAs**

| siRNAs/shRNAs | Sequences (5’-3’) |
| --- | --- |
| LINC01977 shRNA-NC | TTCTCCGAACGTGTCACGT |
| LINC01977 shRNA | GCATTCGTCTTCCCTGGATTT |
| Notch2 shRNA | CCGGAGCGGTGTACCATT |
| RBM39 siRNA-NC | UUCUCCGAACGUGUCACGUTT |
| RBM39 siRNA | GGAACAACUUAAUGGAUUUTT |
| IGF2BP2 siRNA-1 | CATGCCGCATGATTCTTGA |
| IGF2BP2 siRNA-2 | GAACGAACTGCAGAACTTA |

**Supplementary Table 3: The primer sequences for qRT-PCR**

| Primer names | Sequences (5’-3’) |
| --- | --- |
| H-β-actin F | AGCGAGCATCCCCCAAAGTT |
| H-β-actin R | GGGCACGAAGGCTCATCATT |
| H-LINC01977 F | GTCTAACGCAGGGGGAAACA |
| H-LINC01977 R | TATTGCCAACAACTGGCCCT |
| H-GAPDH F | AGAAGGCTGGGGCTCATTTG |
| H-GAPDH R | AGGGGCCATCCACAGTCTTC |
| H-U2 F | ATACGTCCTCTATCCGAGGACA |
| H-U2 R | TGGAGGTACTGCAATACCAGGT |
| H-MEG3 F | GGCCTCTCGTCTCCTTCCT |
| H-MEG3 R | GGGTCCCACATTCGAGGTC |
| H-RBM39 F | AGATGGACAACTGCCTCATTAC |
| H-RBM39 R | GCCTCCCAGTGTTCACATATAC |
| H-Notch2 F | CAACCGCAATGGAGGCTATG |
| H-Notch2 R | GCGAAGGCACAATCATCAATGTT |
| H-IGF2BP2 F | GTTGGTGCCATCATCGGAAAGG |
| H-IGF2BP2 R | TGGATGGTGACAGGCTTCTCTG |

Abbreviations: H, Human; M, Mouse; F, Forward; R, Reverse.

**Supplementary Table 4: Antibodies used in this study**

| **Antibody name** | **Source** | **Item number** |
| --- | --- | --- |
| E-cadherin | proteintech | 20874-1-AP |
| N-cadherin | CST | 13116 |
| Vimentin | CST | 5741 |
| MMP-2 | CST | 40994 |
| MMP-9 | CST | 13667 |
| Ki-67 | abcam | ab16667 |
| CD31 | abcam | ab182981 |
| GAPDH | proteintech | 60004-1-Ig |
| RALY | Novus | NBP2-20065 |
| DHX9 | Novus | NB110-40579 |
| Notch2 | CST | 5732T |
| HEY1 | proteintech | 19929-1-AP |
| HES6 | Abclonal | A16547 |
| IGF2BP2 | abcam | ab124930 |
| m^6^A | abcam | ab208577 |
| HRP mouse antibody | CST | 7074 |
| HRP rabbit antibody | CST | 7076 |
