# Supplementary material for: The cancer-testis lncRNA LINC01977 promotes HCC progression by interacting with RBM39 to prevent Notch2 ubiquitination
Source: Cell Death Discov. 2023 May 18;9:169. doi: 10.1038/s41420-023-01459-1 (PMC10192213; doi:10.1038/s41420-023-01459-1)

Figure 2

Figure 2h

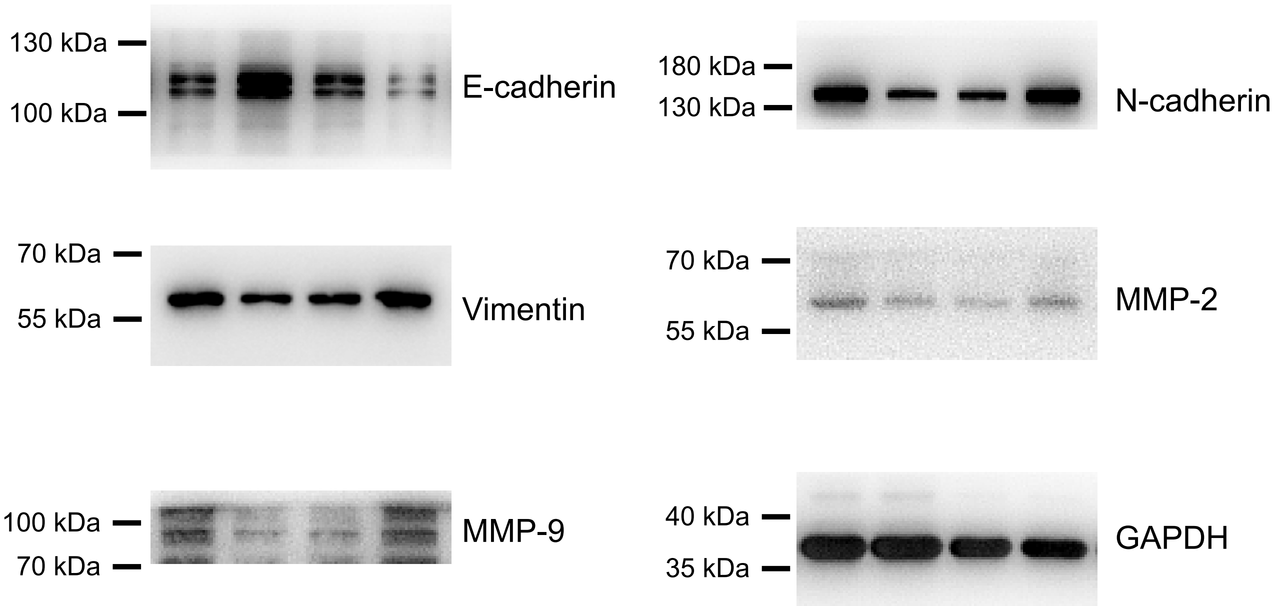

Figure 4

Figure 4f

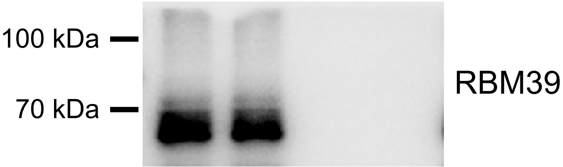

Figure 5

Figure 5d

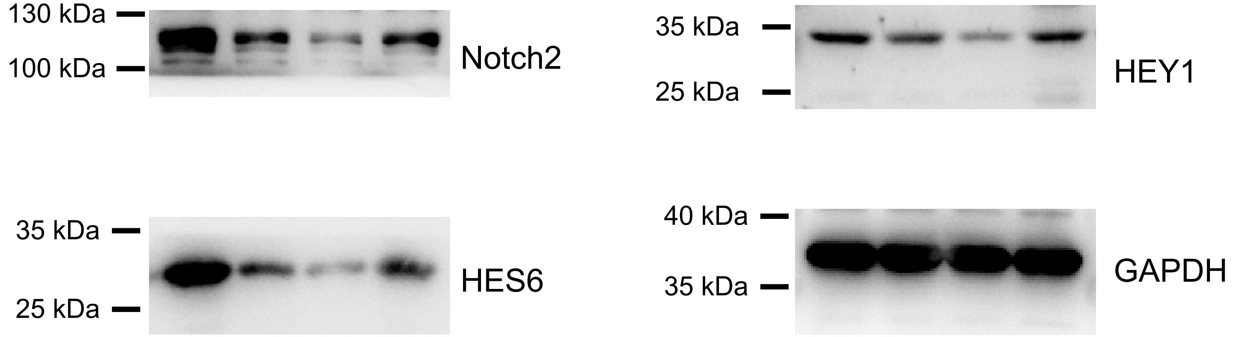

Figure 5g

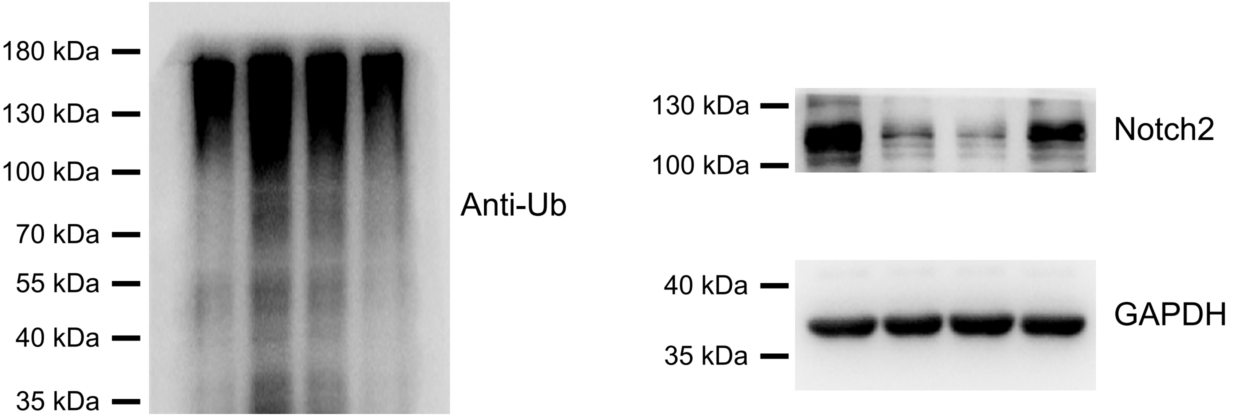

Figure 5h

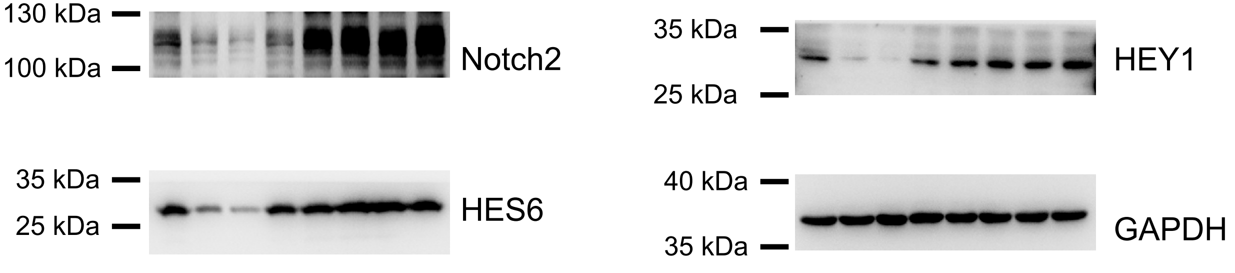

Figure 6

Figure 6a

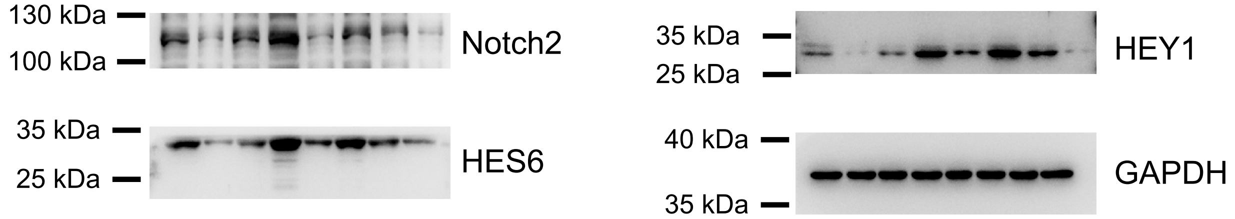

Figure 6b

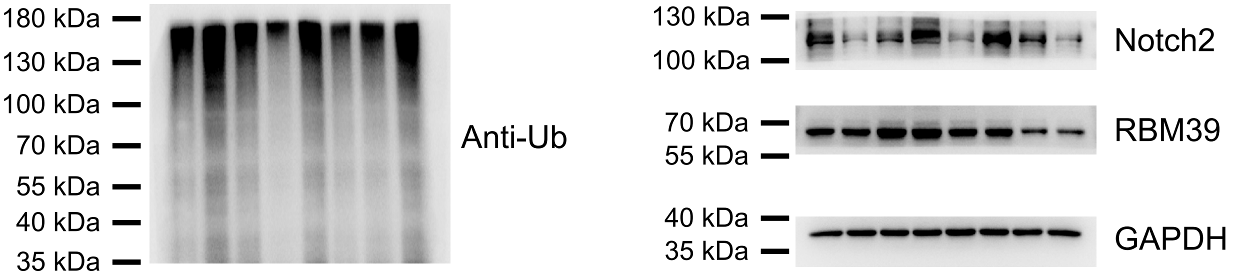

Figure 6e

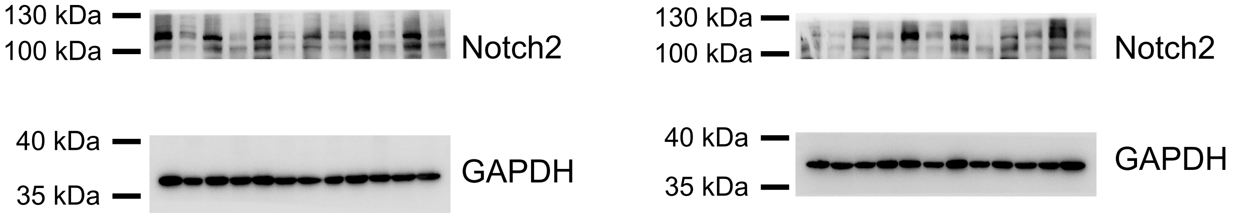

Figure 7

Figure 7b

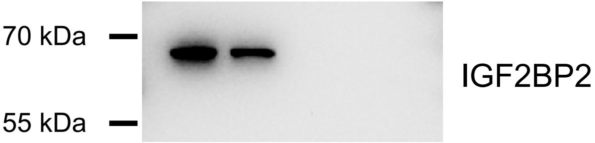

Figure 7e

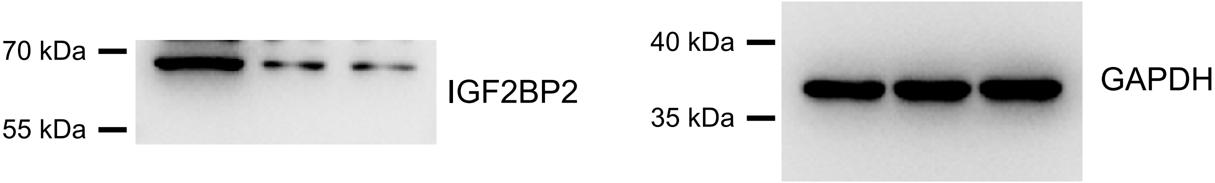

Figure S4

Figure s4c

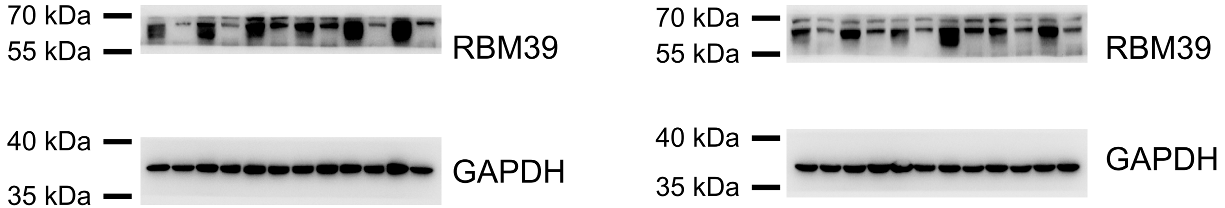

Figure S5

Figure s5c

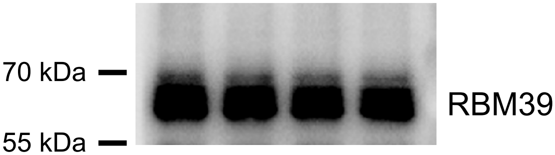

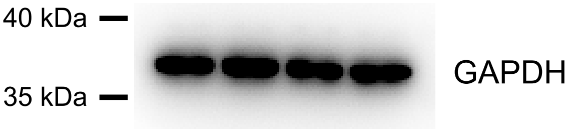

Figure S6

Figure s6c

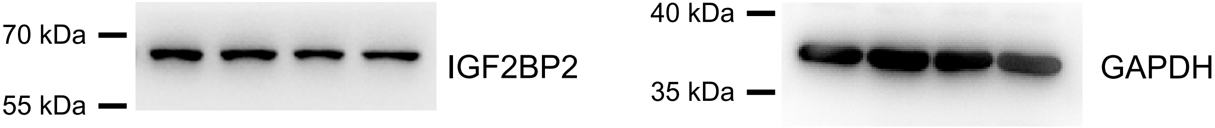

Supplement: Supplementary file 2 — Original Data File [file 41420_2023_1459_MOESM2_ESM.pdf]
